# The Contribution of Working Memory Areas to Verbal Learning and Recall in Primary Progressive Aphasia

**DOI:** 10.3389/fneur.2022.698200

**Published:** 2022-02-17

**Authors:** Alexandros Afthinos, Charalambos Themistocleous, Olivia Herrmann, Hongli Fan, Hanzhang Lu, Kyrana Tsapkini

**Affiliations:** ^1^Department of Neurology, Johns Hopkins School of Medicine, Baltimore, MD, United States; ^2^The Russell H. Morgan Department of Radiology & Radiological Science, Johns Hopkins University School of Medicine, Baltimore, MD, United States; ^3^Department of Biomedical Engineering, Johns Hopkins School of Medicine, Baltimore, MD, United States; ^4^F.M. Kirby Research Center for Functional Brain Imaging, Kennedy Krieger Research Institute, Baltimore, MD, United States; ^5^Department of Cognitive Science, Johns Hopkins University, Baltimore, MD, United States

**Keywords:** working memory, verbal learning, recall, Rey Auditory Verbal Learning Test (RAVLT), primary progressive aphasia (PPA), arterial spin labeling MRI, perfusion imaging, pCASL

## Abstract

Recent evidence of domain-specific working memory (WM) systems has identified the areas and networks which are involved in phonological, orthographic, and semantic WM, as well as in higher level domain-general WM functions. The contribution of these areas throughout the process of verbal learning and recall is still unclear. In the present study, we asked, what is the contribution of domain-specific specialized WM systems in the course of verbal learning and recall? To answer this question, we regressed the perfusion data from pseudo-continuous arterial spin labeling (pCASL) MRI with all the immediate, consecutive, and delayed recall stages of the Rey Auditory Verbal Learning Test (RAVLT) from a group of patients with Primary Progressive Aphasia (PPA), a neurodegenerative syndrome in which language is the primary deficit. We found that the early stages of verbal learning involve the areas with subserving phonological processing (left superior temporal gyrus), as well as semantic WM memory (left angular gyrus, AG_L). As learning unfolds, areas with subserving semantic WM (AG_L), as well as lexical/semantic (inferior temporal and fusiform gyri, temporal pole), and episodic memory (hippocampal complex) become more involved. Finally, a delayed recall depends entirely on semantic and episodic memory areas (hippocampal complex, temporal pole, and gyri). Our results suggest that AG_L subserving domain-specific (semantic) WM is involved only during verbal learning, but a delayed recall depends only on medial and cortical temporal areas.

## Introduction

The ability to learn and remember new information diminishes significantly with aging (1), and it is particularly impaired in neurodegenerative disorders such as mild cognitive impairment, dementia, and primary progressive aphasia (2, 3). Several areas have been implicated in verbal learning and memory. Lesion studies from the time of patient HM (Squire, HM legacy), as well as functional imaging studies, have highlighted the contribution of medial structures, such as the hippocampus, and specific cortical networks, for instance the default mode network (DMN) encompassing temporal, parietal, and frontal areas (4, 5). Ranganath and Richley described the network of areas (cortical and subcortical) involved in verbal learning and memory and highlighted the involvement of these areas in relation to (a) the type of information to be recalled and (b) the timing of the stages of learning (6).

Regarding the type of information to be learned (encoded) and retrieved, previous studies claimed that encoding and retrieval are hemisphere-specific and depend on the type of information: verbal information is encoded in the left middle frontal gyrus (a verbal working memory (WM) area), whereas visuo-spatial information is encoded in the right middle frontal gyrus (a spatial WM area) (7, 8). Recent evidence from transcranial magnetic stimulation (TMS) in healthy controls and from stroke (9–11) confirmed these hemispheric specializations, and the role of WM in the learning phase (encoding of information). However, retrieval of this information mainly requires the recruitment of the hippocampus, whereas the middle frontal gyrus shows the contribution but to a lesser extent (7–10). Regarding the timing and temporal order of the stages of learning, previous studies have concentrated on the areas involved in different stages of encoding vs. retrieval with a particular emphasis on the contribution of WM areas in encoding and learning of new information. Thus, evidence from repetition transcranial magnetic stimulation (rTMS) showed that parietal stimulation interfered with the early encoding phase, whereas middle frontal gyrus stimulation interfered with both encoding and delayed recall phases (12, 13). Of note, the hippocampus cannot be targeted with cortical stimulation techniques such as TMS. Conversely, particular emphasis on the retrieval stage has highlighted the role of different areas such as the anterior temporal system, which is responsible for object recognition and associative memory of objects, and the posterior medial system for the memory of the context of an event, i.e., episodic and source memory (6, 14). Ranganath and Ritchey proposed that the hippocampus enables the link between representations of entities in the anterior mnemonic network and representations of context in the posterior mnemonic network. Furthermore, recent studies suggest that the default mode network and, in particular, the AG, the main hub in DMN, gets activated during a conscious recollection, confirming its role in integrating or attending to contextual information retrieved *via* the hippocampus (15, 16).

To determine the temporal order of areas involved in learning and recall, several studies have used standardized verbal learning and memory tests such as the Rey Auditory Verbal Learning Test (RAVLT) or the California Verbal Learning Test (CVLT), and other equivalents, in which, an individual is asked to memorize a list of 12–15 words over several trials (usually five) and then the same word-list is tested in a delayed recall (usually intersected with an interference list) (17). In support of different networks involved in verbal learning vs. recall of information, volumetric MRI studies (18–20) of patients with dementia showed that Trial 1 scores of RAVLT correlated with inferior parietal, middle frontal gyrus, and temporal pole. However, as learning occurred, correlations were stronger between Trial 5 and medial temporal lobe (hippocampal complex) and temporal pole volumes, and delayed recall scores correlated only with hippocampal volume (18–20). Trial 1 of RAVLT is also considered a supraspan measure that reflects sensory attentional component, with negligible correlation with learning measures (21). While the fundamental role of WM in the learning phase of new information has been well established since Baddeley's model (22, 23), the nature and characteristic of the information are temporarily withheld in WM buffers, such as the phonological loop, was not characterized until recently. Only few studies have looked at the role of WM buffers beyond phonological encoding of verbal information, e.g., semantic information (24, 25).

Recent neuropsychological evidence has given rise to domain-specific (i.e., phonological, semantic, or orthographic) vs. domain-general approaches to WM. These models assume that each domain contains its own set of “WM” systems dedicated to the maintenance and manipulation of different types of information such as phonological, semantic, or orthographic (26, 27), but refer to alternative views by Logie, Camos, and Cowan (28). Evidence for the existence of domain-specific temporary information buffers comes from the neuropsychological (mostly in the post-stroke literature) and the neuroimaging literature (in healthy controls and patients). Domain-specific information buffers have been identified for: (a) phonological information subserved by inferior parietal lobe (IPL) regions and, in particular, the left supramarginal gyrus (SMG) (23), (b) orthographic information subserved by adjacent inferior parietal areas and, in particular, the posterior part of the left SMG or anterior part of the angular gyrus (AG) (27), and (c) semantic information subserved by posterior inferior parietal areas and, in particular, the AG [refer to (27) for a recent review]. These areas have been considered the loci of phonological WM, orthographic WM, and semantic WM, respectively. The term “WM” in most of these models is used to denote a temporary hold of specific information, a type of short-term memory buffer, rather than a system comprising executive control over this information (e.g., monitoring, manipulation, and other control processes), functions ascribed rather to middle frontal and superior parietal gyri (29–32). For these more executive WM operations, a plethora of evidence shows that they are performed in frontal areas, and, in particular, the left middle frontal gyrus (MFG) and MFG_dorsolateral prefrontal cortex (DLPFC), or other more superior parietal areas devoted to attention such as the left inferior parietal sulcus (29–31, 33, 34). However, it is still unclear what type of domain-specific WM systems are necessary for verbal learning and recall of learned information and what the contribution and interplay are between frontal, parietal, and medial temporal areas during the process of learning and recall. The present study aimed to bridge this gap in our knowledge by probing the role of domain-specific and domain-general WM areas in verbal learning and memory using evidence from MR-perfusion imaging in patients with primary progressive aphasia (PPA).

Primary progressive aphasia (PPA) is a neurodegenerative disorder affecting primarily language functions of the left hemisphere. There are three PPA variants, the non-fluent variant (nfvPPA) with deficits mainly in grammar and speech production, the logopenic variant (lvPPA) with deficits mainly in naming and repetition of words and sentences, and the semantic variant (svPPA) with deficits in object naming and comprehension (35, 36). The nature of deficits is associated with the location of brain atrophy, hypoperfusion, or hypometabolism (35, 37–40). In recent classification, approaches using machine learning methodologies, we and others were able to distinguish the three main PPA variants with comparable to clinicians' accuracy using parts-of-speech measurements (41, 42). In addition to language deficits, all three PPA variants exhibit impairments in verbal episodic and WM (2, 3). In particular, a recent meta-analysis of 41 studies indicated that verbal learning, assessed by verbal episodic memory tests, such as the RAVLT (43) and WM, assessed by tests such as digit span backward, are significantly impaired in all three PPA variants compared to healthy controls (2). All patients with PPA had substantially worse episodic and WM than healthy controls.

Early studies in PPA were primarily concerned with reporting correlations between brain areas and language tasks since PPA is concerned a language syndrome. Recent accounts, however, have documented verbal episodic memory and learning deficits in all three PPA variants, and associated these with WM deficits (2). In our previous transcranial direct current stimulation (tDCS) clinical trial, we also found that areas involved in verbal learning predicted treatment outcomes. We evaluated which brain areas are critical for response to tDCS (44). We used the volumetric regions of interest (ROI) approach to identify the anatomical areas whose volumes have significantly predicted the additional tDCS effects on written naming/spelling. For trained words, where learning during therapy was involved, the volumes of the left AG and left PCC predicted additional tDCS gains. These findings show that areas involved in either or both semantic integration and episodic memory contribute to the maintenance of training effects. In a subsequent study (45), we prompted the cognitive predictors of written naming/spelling improvement due to tDCS vs. written naming/spelling alone and found that an important predictor for spelling words was RAVLT scores (5 trials sum), i.e., the performance to learning words in context (lists). However, there have been no studies to systematically examine the contribution of brain areas through all stages of verbal learning in PPA.

This article addresses an important topic concerning the role of different brain areas in verbal learning and recall in PPA. We modeled the perfusion (relative cerebral blood flow, rCBF) of language and memory areas of the left and right hemispheres on each learning trial, namely Trial 1 to Trial 5, then total recall, i.e., the sum of trials to measure the sum of learning according to the literature, and delayed recall. We used pseudo-continuous arterial spin labeling (pCASL) as this MR image analysis provides spatial resolution and non-contrast measurement of rCBF (46, 47). Based on the above-reviewed literature on phonological and semantic WM, we hypothesized that: (a) domain-specific WM (phonological and/or semantic depending on the encoding) will be significantly involved in the initial stages of learning since both types of encoding and temporary storage are needed for subsequent learning and recall; (b) the role of domain-specific WM will diminish after learning has taken place, i.e., at the recall stage from long-term memory; (c) the hippocampus and other semantic temporal regions will be significantly involved in delayed recall, given the documented role of episodic and semantic memory at recall, e.g., effects of concreteness, categorical organization (6, 48).

## Materials and Methods

### Participants

In total, 32 patients (15 women and 17 men) with PPA participated in the study: 13 with lvPPA, 14 with nfvPPA, and five with svPPA variant. Diagnosis of PPA was provided by a neurologist(s) according to consensus criteria (35). Data of the participants were collected as part of a clinical trial conducted at Johns Hopkins University [NCT02606422; (49)]. Patients had progressive speech and language deficits primarily without major deficits in other cognitive domains or developmental or nondegenerative neurological disorders (e.g., stroke). The participants completed a series of neuropsychological assessments which were followed by MRI. Demographics and baseline values of neuropsychological assessments are presented in Table 1. All patients were native English speakers, with normal or corrected vision, and a minimum of high school education. Participants provided informed consent and the study was approved by the Johns Hopkins Hospital Institutional Review Board.

**Table 1:** Demographic and neuropsychological data of the participants.

	***M***	***SD***
Age	66.53	5.96
Education	16.62	2.09
Total severity (FTD-CDR)	5.88	4.72
Language severity (FTD-CDR)	1.75	0.85
Digit span forward	4.50	1.77
Digit span backward	3.08	1.58
RAVLT trial 1 (out of 15)	3.13	2.52
RAVLT trial 2 (out of 15)	4.97	3.32
RAVLT trial 3 (out of 15)	5.81	3.52
RAVLT trial 4 (out of 15)	6.19	3.53
RAVLT trial 5 (out of 15)	6.90	4.34
Verbal learning (RAVLT Trial 5-1)	3.71	3.17
RAVLT total recall (sum of Trial 1 to Trial 5; out of 60)	27.32	16.13
Recall (RAVLT delayed recall; out of 15)	5.41	3.86

*RAVLT, Rey Auditory Verbal Learning Test (43, 50, 51)*.

*Digit span forward and backward refers to the spans from Wechsler Adult Intelligence Scale III (52). M represents the mean and SD the standard deviation*.

### Neuropsychological Evaluation (RAVLT)

Participants completed the RAVLT at baseline (43, 53). In short, they were verbally presented with a list of 15 words (one word per second) which they had to recall immediately following the presentation. Presentation and recall of the same original list were repeated 5 times (Trials 1–5). Subsequently, participants were presented with an alternate list which they had to recall only once (interference—Trial 6). Then, they were asked to recall as many words as they could from the original list (free recall—Trial 7). Following a 25-min break, they were asked again to recall as many words as they could from the original list (delayed recall—Trial 8). In this report, we present the results on Trial 1–5 as well as the total recall (sum of Trials 1–5) and Trial 8 (delayed recall) because we intended to probe the areas involved in verbal learning and memory and not interference or other functions.

### Magnetic Resonance Imaging

Baseline MRI experiments were performed immediately after the neuropsychological evaluation on a 3 Tesla MRI scanner using an 8-channel head coil (Philips Healthcare, Best, Netherlands). All participants were requested to refrain from consuming caffeine and alcohol for 8 h prior to the MRI scans. A body coil was used for radiofrequency (RF) transmission. Foam padding was placed around the head to minimize motion during MRI scan acquisition. The MRI protocol consisted, among other sequences, of a T1-weighted magnetization-prepared rapid acquisition of gradient echo sequence (T1-MPRAGE) and a pCASL sequence (54). The scan parameters of the T1-MPRAGE sequence were as follows; TR/TE/TI = 8.1/3.7/1,100 ms, shot interval 2,100 ms, flip angle = 12°, voxel size 1 × 1 × 1 mm^3^, number of slices 160, sagittal slice orientation, and duration 3 min 57 s. The PCASL MRI is the recommended method in the clinic by the International Society for Magnetic Resonance in Medicine (ISMRM) perfusion study group and the European consortium for ASL in dementia (55). Scan parameters of the pCASL sequence were: field of view (FOV) = 205 × 205 mm^2^, matrix = 64 × 64, 39 axial slices, thickness = 3.2 mm, TR/TE = 5,817/9.3 ms, labeling duration = 1.8 s, post labeling delay = 1.8 s, 6 pairs of label and control images, and 3D gradient-and-spin-echo (GraSE) with background suppression, with duration 5 min 14 s. A separate M0 scan was also required using the following parameters: TR = 10,000 ms, duration 1 min, and 10 s.

### Cerebral Blood Flow (CBF) and Brain Volume Quantification

CBF maps were generated from the pCASL MRI images using the Johns Hopkins University's cloud based ASL analysis software, ASL-MRICloud (https://braingps.mricloud.org/asl) (56) and_ENREF_19. All ASL scripts on the cloud server were written in MATLAB 2013 and SPM12. The analysis procedure followed the recommendations in the ASL white paper (55).

The ASL-MRICloud data processing used the following procedures. Motion correction was performed and the difference between control and label image pairs (control—label) was calculated. Quantification of CBF in physiological units (ml/100 g/min) was based on a kinetic model (55):


(1) $$\begin{array}{r} CBF=\frac{6000\cdot S_{ASL}\cdot \lambda \cdot e^{w/T_{1blood}}}{2\alpha \cdot T_{1blood}\cdot M_{0}\cdot \left(1-e^{-\tau /T_{1blood}}\right)} \end{array}$$

where *S*_*ASL*_ is the signal difference between the control and label images from the pCASL acquisition; λ is the brain/blood partition coefficient, assumed to be 0.9 ml /g; w is the post-labeling delay time (1,525 ms); *T*_1*blood*_ is the longitudinal relaxation time of blood and was set at 1,650 ms; α is the labeling efficiency, assumed to be 0.85; M0 reflects the signal intensity of spins at equilibrium magnetization and was estimated from the M0 scan; and τ is the label duration (1,650 ms). The CBF map was co-registered to the T1-weighted MPRAGE image by means of a 12-parameter affine transformation.

Using MRIcloud (57, 58), a cloud-based platform that performs automated image mapping and segmentation, the T1 data were normalized to Montreal Neurologic Institute (MNI) template, in which 19 brain atlases were used to transform the individual image to the template and subsequently, the warped images were automatically segmented into 289 brain regions. The use of multiple atlases reduces errors produced by individual atlas-based image registration. Brain volumes were then automatically extracted using MRIcloud from the brain regions of interest mentioned below.

Relative CBF was calculated by dividing the CBF within a brain region with the value of CBF over the entire brain. Previous studies have shown that the use of relative CBF, as opposed to absolute CBF, is useful in reducing global confounding factors, e.g., breathing pattern, on the regional perfusion assessment (59).

### Statistical Analyses

To determine which areas predict verbal learning and recall, i.e., performance on RAVLT's Trial 1, Trial 2, Trial 3, Trial 4, Trial 5, total recall (sum of Trial 1 through Trial 5) and Delayed Recall (Trial 8), we regressed the performance on each of these outcomes on the perfusion (CBF) of 25 pre-selected verbal learning and memory areas in each hemisphere. These areas are as follows: Angular Gyrus (AG), Fusiform Gyrus (FuG), Hippocampus (Hippo), Inferior Frontal Gyrus Pars Opercularis (IFG Opercularis), Inferior Frontal Gyrus Pars Orbitalis (IFG Orbitalis), Inferior Frontal Gyrus Pars Triangularis (IFG Triangularis), Insula (Insula), Inferior Occipital Gyrus (IOG), Inferior Temporal Gyrus (ITG), Lateral Fronto-Orbital Gyrus (LFOG), DPFC Middle Frontal Gyrus Dorsolateral Prefrontal Cortex (MFG), Middle Frontal Gyrus (MFG), Middle Fronto-Orbital Gyrus (MFOG), Middle Occipital Gyrus (MOG), Middle Temporal Gyrus (MTG), Middle Temporal Gyrus Pole (MTG Pole), Posterior Cingulate Cortex (PCC), Parahippocampal Gyrus (PHG), Pre-Cuneus (Prcu), Superior Frontal Gyrus (SFG), Supramarginal Gyrus (SMG), Superior Parietal Gyrus (SPG), Superior Temporal Gyrus (STG), Superior Temporal Gyrus Pole (STG Pole), and Thalamus.

Subsequently, we employed the stepwise regressions on all brain areas, based on the cross-validation R-square. The first step starts from the model with no predictor areas (say model_0_), and finds the area, say Area _model0 → 1_ that, when included into the model, gives the largest increase Δ(R^2^)_model0 → 1_, in the cross-validated R^2^, than if any other area were included instead. We choose a threshold of 1% so that if this Δ(R^2^)_model0 → 1_ is >1% than we include that area, Area _model0 → 1_, in the more accurate new model, say model_1_. Each next step continues similarly to find if there is an area, among those that are not yet included in the model, that would produce the largest (and >1%) increase in R^2^. An implication of using the increase in cross-validated R^2^ as an effect size is that it is a reliable indicator of the relative importance of the predictions in a model (Refer to Tables 2–**8** for Trials 1, 2, 3, 4, 5, Total recall and Delayed Recall). The final model from that selection was evaluated using a global *F*-test on the subset group of brain areas selected by the stepwise regression. **Table 9** reports the *F* statistic and the corresponding *p-*value for the hypothesis of having them all in the regression vs. not having these areas in the model. The statistical analysis was conducted in R (R Core Team 2), using built-in routines and statistical packages for stepwise regression that we had previously employed (44, 60).

**Table 2:** Results from a stepwise regression with the brain areas as predictors, and Trial 1 as the dependent variable.

	**Index**	**R2.cum**	**R2.increase**	**MSE**
No predictors	NA	0.0000	NA	7.162
STG left*	23	0.1952	0.19522	5.764
Hippo left	3	0.2316	0.03634	5.504
SFG right	45	0.2550	0.02340	5.336

*The table shows the cumulative increase of the R^2^ (R2.cum) as variation explained by the model, the R^2^ increase (R2. increase) added to the model, and the mean squared error (MSE). Asterix indicates a 0.05 R2 increase. Asterix indicates a 0.05 R^2^ increase*.

## Results

### Immediate Recall (Trial 1)

Immediate recall refers to the items recalled immediately after the first presentation of the first word list in RAVLT (Trial 1). Investigating the independent contribution of 36 brain regions to RAVLT Trial 1, we found that the perfusion of the left STG explained most of the variance on Trial 1 (19.5%, refer to Table 2). Here and in the subsequent tables, the increase of R^2^ is provided in brackets as a percent score. The other two areas explained <1% of the variance that was our significance threshold. These findings indicate that immediate recall depends mainly on acoustic processing (as indicated by the contribution of left STG). The overall model with the three predictors was significant at *p* = *0.0*004 over the models which do not have these (refer to **Table 9**).

### Verbal Learning (Trials 2–5)

Trials 2–5 refer to items recalled in each consecutive trial of the same wordlist, i.e., verbal learning over each Trial, following the first introduction of the wordlist in Trial 1. As shown in Tables 3–6, perfusion of the left AG explained most of the variance on all Trials: Trial 2 (44%, refer to Table 3; *p* = 0.000043, refer to **Table 9**), Trial 3 (37%, refer to Table 4; *p* = 0.000000042, refer to **Table 9**), Trial 4 (38%, refer to Table 5; *p* = 0.000000047, refer to **Table 9**), and Trial 5 (41%, refer to Table 6; *p* = 0.000011, refer to **Table 9**). These findings indicate that verbal learning through Trial 5 depends mainly on the left AG. Other areas that become increasingly involved as learning progress are the areas subserving lexical-semantic processes (left ITG and left FuG, Table 4; left STG pole, Table 6; mnemonic process (left PHG, Table 4; left hippocampus, and Table 5; domain-general WM (right MFG, Table 4; left MFG, Table 6).

**Table 3:** Results from a stepwise regression with the brain areas as predictors and Trial 2 as the depended variable.

	**Index**	**R2.cum**	**R2.increase**	**MSE**
No predictors	NA	0.0000	NA	11.609
AG left*	1	0.4369	0.43694	6.537
MTG left	15	0.4541	0.01721	6.337

*The table shows the cumulative increase of the R^2^ (R2.cum) as variation explained by the model, the R^2^ increase (R2. increase) added to the model, and the MSE. Asterix indicates a 0.05 R^2^ increase*.

**Table 4:** Results from a stepwise regression with the brain areas as predictors, and Trial 3 as the dependent variable.

	**Index**	**R2.cum**	**R2.increase**	**MSE**
No predictors	NA	0.0000	NA	13.467
AG left*	1	0.3690	0.36897	8.498
SPG right	47	0.3975	0.02851	8.114
PHG left*	18	0.4769	0.07937	7.045
Hippo left	3	0.5109	0.03400	6.587
ITG left*	9	0.5606	0.04976	5.917
FuG left*	2	0.6253	0.06469	5.046
MFG right*	37	0.7402	0.11488	3.499
IFG opercularis right	29	0.7690	0.02879	3.111

*The table shows the cumulative increase of the R^2^ (R2.cum) as variation explained by the model, the R^2^ increase (R2. increase) added to the model, and the MSE. Asterix indicates a 0.05 R^2^ increase*.

**Table 5:** Results from a stepwise regression with the brain areas as predictors, and Trial 4 as the depended variable.

	**Index**	**R2.cum**	**R2.increase**	**MSE**
No predictors	NA	0.0000	NA	13.344
AG left*	1	0.3811	0.38111	8.258
IFG orbitalis Left	5	0.4074	0.02632	7.907
LFOG left*	10	0.4654	0.05799	7.133
IOG left	8	0.5043	0.03888	6.614
ITG left	9	0.5411	0.03679	6.124
Hippo left*	3	0.6603	0.11921	4.533
SPG right	47	0.6909	0.03061	4.124
Hippo right	28	0.7068	0.01593	3.912
MFG left	12	0.7318	0.02497	3.579
PCC left	17	0.7587	0.02691	3.220
Thalamus right	50	0.7706	0.01187	3.061

*The table shows the cumulative increase of the R^2^ (R2.cum) as variation explained by the model, the R^2^ increase (R2. increase) added to the model, and the MSE. Asterix indicates a 0.05 R^2^ increase*.

**Table 6:** Results from a stepwise regression with the brain areas as predictors, and Trial 5 as the dependent variable.

	**Index**	**R2.cum**	**R2.increase**	**MSE**
No predictors	NA	0.0000	NA	20.352
AG left*	1	0.4118	0.41183	11.970
IFG orbitalis left	5	0.4422	0.03033	11.353
STG left pole*	24	0.5041	0.06199	10.091
MFG left*	12	0.5593	0.05513	8.969

*The table shows the cumulative increase of the R^2^ (R2.cum) as variation explained by the model, the R^2^ increase (R2. increase) added to the model, and the MSE. Asterix indicates a 0.05 R^2^ increase*.

### Sum of Learning (Sum of Trials 1–5)

Total recall refers to the Sum of Learning of all RAVLT learning trials (Trial 1–Trial 5), and it is an indication of processes used throughout the learning part of the assessment. As shown in Table 7, perfusion of the left AG explained most of the variance in the total amount of learning that has taken place (42%, refer to Table 7; *p* = 0.0000000058, refer to **Table 9**). Other significant areas explaining above 5% of variance were the left ITG (11%), the left PHG (9%), and the right MFG (7%). These findings indicate that the left AG is the area with the highest contribution for the sum of learning that takes place in the five consecutive trials. They also show that for learning to take place, a network of areas subserving a domain-specific (semantic) WM (left AG), lexical-semantic processes (left FuG and left ITG), and domain-general WM (right MFG), as well as mnemonic processes (left PHG), are needed.

**Table 7:** Results from a stepwise regression with the brain areas as predictors and Sum of Learning (Total RAVLT) score as the dependent variable.

	**Index**	**R2.cum**	**R2.increase**	**MSE**
No predictors	NA	0.0000	NA	276.08
AG left*	1	0.4183	0.41834	160.59
FuG left	2	0.4450	0.02667	153.23
ITG left*	9	0.5590	0.11404	121.74
MFG right*	37	0.6255	0.06641	103.41
PHG left*	18	0.7192	0.09376	77.52
SPG right	47	0.7679	0.04872	64.07
Insula right	32	0.8028	0.03486	54.44

*The table shows the cumulative increase of the R^2^ (R2.cum) as variation explained by the model, the R^2^ increase (R2. increase) added to the model, and the MSE. Asterix indicates a 0.05 R^2^ increase*.

### Delayed Recall (Trial 8)

Delayed recall refers to the number of words recalled 25min after the last immediate recall learning trial (Trial 5). As shown in Table 8, the areas which significantly increased the model's R^2^ exclusively are the areas subserving semantic and mnemonic processes, i.e., the left MTGpole (18%), left MTG (18%), left IFG orbitalis (6%), and left insula (6%) along with the left hippocampus (17%) (refer to Table 8, *p* = 0.0000031, refer to Table 9). Importantly, the left AG, which subserves semantic WM and is the most important areas during learning, had no contribution in delayed recall. See summary Table 10 for a summary of results.

**Table 8:** Results from a stepwise regression with the brain areas as predictors and Delayed Recall (Trial) as the dependent variable.

	**Index**	**R2.cum**	**R2.increase**	**MSE**
No predictors	NA	0.0000	NA	15.938
MTG left pole*	16	0.1790	0.17898	13.086
Hippo left*	3	0.3496	0.17066	10.366
IFG orbitalis left*	5	0.4102	0.06055	9.400
MTG left*	15	0.5901	0.17992	6.533
Insula left*	7	0.6516	0.06147	5.553
IFG opercularis Left	4	0.7014	0.04980	4.760
FuG left	2	0.7142	0.01287	4.554

*The table shows the cumulative increase of the R^2^ (R2.cum) as variation explained by the model, the R^2^ increase (R2. increase) added to the model, and the MSE. Asterix indicates a 0.05 R^2^ increase*.

**Table 9:** The table provides the F value and the corresponding p-value from the model comparison of the estimated models from the stepwise regression, and with a model without those predictors (intercept 1); Residual Degrees of Freedom (Res.DF), residual sum of squares (RSS), Degrees of Freedom (DF); Sum of squares (Sum of Sq), F statistic (F), *p*-value; significance codes: 0 ‘^***^' 0.001 ‘^**^’ 0.01 ‘^*^’ 0.05 ‘.’ 0.1 ‘ ’ 1.

	**Res.Df**	**RSS**	**Df**	**Sum of Sq**	**F**	***p*** **value**	
Trial 1	32	222					
	29	141	3	80.8	5.53	0.004	**
Trial 2	32	360					
	30	184	2	176	14.3	<0.0001	***
Trial 3	32	418					
	24	63	8	355	16.8	<0.0001	***
Trial 4	32	414					
	24	63	8	351	16.6	<0.0001	***
Trial 5	32	632					
	28	237	4	394	11.6	0.000011	***
Delayed recall	27	415					
	20	74	7	341	13.1	<0.0001	***
Sum of learning	32	8,567					
	25	1,253	7	7,314	20.9	<0.0001	***

**Table 10:** Summary table for all regression models.

**Trials**	**Trial 1**	**Trial 2**	**Trial 3**	**Trial 4**	**Trial 5**	**Sum of learning**	**Delayed recall**
AG left		AG	AG	AG	AG	AG	
MFG left/right			MFG right		MFG left	MFG right	
IFG left							IFG orb
Insula left							Insula
LFOG left				LFOG			
STG left	STG						
STG pole left MTGpole left					STG pole		MTGpole
MTG left							MTG
ITG/FuG left			ITG/FuG			ITG	
Hippo/PHG left			PHG	Hippo		PHG	Hippo

## Discussion

In the present study, we used perfusion (from PCASL-MRI) to investigate which brain areas contribute to verbal learning and recall on a widely used test of verbal learning, the RAVLT (43), in a group of patients with PPA. We asked how domain-specific (phonological and semantic) WM and domain-general WM areas are involved over the course of learning by using data from the consecutive word-list learning trials of the RAVLT. We showed that in the initial stage (immediate recall, Trial 1), verbal learning depends on the left STG subserving phonological processing, and, in particular, acoustic encoding (61). As learning progresses (Trials 2–5), verbal learning relies predominantly and heavily on the left AG subserving domain-specific (semantic) WM (27, 62). However, other areas become involved as well, such as the left temporal cortex (ITG/FuG/MTGpole/STGpole), subserving lexical and semantic storage (63), and the left hippocampus, and PHG subserving mnemonic encoding (5, 64). In addition to the left AG and the whole semantic network, verbal learning involves domain-general WM areas (left MFG). Finally, the left AG, the most important area during learning, is not at all involved in the delayed recall of learned information. Delayed recall relies exclusively on areas of the semantic and mnemonic network, such as the left MTG and MTGpole responsible for semantic storage (65–67), the left hippocampus responsible for mnemonic encoding and storage, and the left IFG orbitalis and insula, responsible for strategic retrieval of semantic information (68, 69) See summary Table 10. The present study allowed us to investigate, for the first time, the progress of verbal learning and the corresponding involvement of brain areas in patients with PPA.

### Early vs. Late Stages of Verbal Learning and Recall

Our results highlight the significant contribution of domain-specific WM, and in particular semantic WM during verbal learning but not recall, and align well with recent evidence on the role of the left AG in semantic WM (27, 62). Further, they emphasize the pivotal role of semantic WM early in verbal learning. The notion that phonological WM is required for learning has been around since Baddeley's early model where phonological encoding through articulatory rehearsal was revealed to be pivotal for learning (22), but the role of semantic WM for learning has been explored only recently (15, 27). Our finding, the left STG is involved in RAVLT Trial 1, confirms the requirement for phonological encoding of new verbal information in the left STG (61) during the early stages of learning. Importantly, the present study highlights the role of the left AG as the main area responsible for the temporary hold of semantic information and its online processing (6, 48). The left AG functions as a semantic buffer, as recently claimed (27, 62), where semantic encoding may take place (15).

Also, the contribution of domain-general WM areas, such as the left MFG in the course of learning shows the important involvement of a fronto-parietal network as learning progresses. Previous studies have highlighted the role of the left MFG in domain-general WM, and, in particular, in the monitoring of information (23, 32) that could be hemisphere-specific depending on the modality, i.e., verbal information in the LH vs. visuospatial information in the RH (9–11, 70). It has also been shown that areas subserving the fronto-parietal domain-general WM network are needed for learning to take place [refer to (19, 71)]. In our previous study (72), we identified specific impairments of this network in patients from all three PPA variants, with a loss of important connectivity hubs in superior frontal and parietal areas and new hubs in the left MFG (72, 73). This aligns with the finding that all PPA variants are impaired in both WM and verbal learning (2, 3). Importantly, the left AG and the left MFG are structurally connected through the dorsal part of the superior longitudinal fasciculus (SLF II) (70). These connections would allow for the use of monitoring and control functions in semantic information that is held online to reinforce the implementation of computations such as semantic integration (15).

As learning progresses, we found that the involvement of semantic processing areas in the left temporal cortex becomes more prominent, indicating that successful learning relies on semantic processing of information, and involves both a temporary hold of semantic relations (in the left AG), as well as storage of lexical/semantic information (in the left MTG, MTGpole, ITG, and FuG) (63) and mnemonic encoding in hippocampal and parahipocampal gyri (5). It has been claimed that within the left temporal lobes, the middle temporal structures (and the pole, in particular) store modality-independent semantic information (63, 66, 74, 75), whereas the left ITG and FuG store modality-specific, orthographic and phonological, representations of words (63, 66, 67, 76). Thus, our results highlight the importance of the AG for verbal learning and add to the growing evidence of its pivotal role in learning due to its structural and functional connections with mnemonic processing areas such as the hippocampal complex, lexical/semantic storage areas such as middle and inferior temporal gyri, and temporal poles (4, 6, 77), as well as its connections with frontal executive areas for monitoring and control, such as the MFG and IFG (32, 63).

The contribution of semantic storage and mnemonic processes becomes more prominent at the stage of a delayed recall. Recall of the learned information (Delayed Recall) depends exclusively on a lexical/semantic network (the left MTG, left MTGpole, left IFG orbitalis, and the left insula), as well as on mnemonic processing in the left hippocampus, but the left AG is no longer needed. This lexical-semantic network is subserved by (a) left MTG pole where amodal semantic representations are stored (65, 66, 74), (b) the left MTG also involved in semantic storage (63), and (c) the left IFG orbitalis, which is involved in selective semantic retrieval (33, 37). In addition, the lack of left MFG involvement in delayed recall aligns with an earlier study showing that there is no significant difference in the memory recall rates between significantly and minimally impaired executive dysfunction groups (78). Importantly, the left AG, where semantic representations are temporarily held (6, 27, 48), was the most important area during the learning but was not at all involved in a delayed recall of learned information.

### The Role of the Left AG: Semantic WM or Episodic Memory?

Several functional roles have been ascribed to the left AG. Beyond serving as a temporary buffer of semantic WM, as recently claimed (27, 62), its involvement has been also claimed to signify other functions both within the semantic system, e.g., for semantic integration (15), and/or for episodic memory and encoding (6). We further discuss how the present results may help disambiguate the role of the left AG as a semantic WM area (buffer for semantic information) vs. its potential role in episodic memory.

One could argue that the involvement of the left AG in verbal learning trials of RAVLT but not in delayed recall in the present study, indicates that the left AG is an area of semantic WM and acts as a semantic buffer rather than an area of episodic memory. On the other hand, each trial may be construed as one episode (event) that needs to be encoded and the words in the list may be construed as the components of this episode. For successful list learning, it is rather the semantics of the words (components) that need to be encoded, and not (necessarily) the sequence and order of words that need to be learned. Therefore, the role of the left AG in verbal episodic memory is related to its role in semantic encoding and temporary hold of semantic information. Thus, semantic encoding is needed for verbal information to be remembered. The left AG is exceptionally located to play this role in the association cortex, being connected, functionally and structurally, with ventral temporal areas where semantic information resides, as well as with medial temporal areas (hippocampal complex), where mnemonic encoding and discrimination takes place (5, 6). The present results of the involvement of AG during verbal learning but not recall suggest that it is important for semantic contextual encoding. They further suggest that contextual (episodic) encoding of verbal information is semantic in nature and depends on the capacity of this semantic buffer. This interpretation would align well with both the attribution of the AG as a semantic WM buffer that temporarily holds semantic information (27), as well as neuromodulation findings of improving semantic binding between a noun and an adjective (15).

Another possibility to accommodate findings of the AG being involved in episodic memory is that this is a multicomponent area in which different subdivisions are responsible for different computations. Using a region of interest rather than a voxel approach, the present study, cannot make any specific claims about AG subareas subserving different functions. The well-known correlation of parietal lobes with both spatial and temporal aspects of information, as well as the large size of the left AG, does not exclude this possibility. However, for such interpretation to be upheld, one would need to consider whether there is evidence of left AG involvement in episodic but not semantic buffering of verbal information. This kind of evidence exists for phonological and orthographic information sequencing but the area seems to be the left SMG, not the left AG (23, 79).

### Limitations

There are certain limitations in the present study. The most important is that the perfusion data may not correspond completely to the previous glucose metabolic brain patterns since the overlap between the two methods is not complete (80). This is explained by the fact that the MRI head coils with multiple receiver channels result in a better signal-to-noise ratio in the cortex than in deeper brain structures (81). Therefore, perfusion in medial brain structures such as the hippocampus may be higher than our estimates. The other limitation of the present study is the sample size. Although the sample of 32 participants with PPA is not a small number given the rarity of the condition, a larger sample size could strengthen the present findings and could also enable us to ask the question of whether different perfusion patterns characterize each PPA variant.

## Conclusions

Our findings on verbal learning and memory measures in PPA show that pCASL is a promising technique that could be developed further as an additional MRI measurement that provides a quick, non-invasive and much less expensive alternative to positron emission tomography (PET) imaging. Our results on verbal learning and delayed recall have implications for a deficit-specific approach to learning and memory interventions. While research on learning and memory disorders has been advancing rapidly, successful treatments with long-term outcomes are non-existent. Neuromodulation through transcranial electrical or magnetic stimulation (tDCS or TMS) shows promise as these techniques could enhance neuroplasticity in areas that are important for learning and memory (82, 83). In particular, the present findings on the role of the left AG in verbal learning suggest that this area could be a particularly important area to target with neuromodulation in order to enhance the verbal learning ability in patients with PPA.

## Data Availability Statement

The raw data supporting the conclusions of this article will be made available by the authors, without undue reservation.

## Ethics Statement

The studies involving human participants were reviewed and approved by Johns Hopkins Medicine Institutional Review Board (IRB#: NA_00071337). The patients/participants provided their written informed consent to participate in this study.

## Author Contributions

KT and HL designed the study. KT, CT, HL, and AA interpreted the data. CT performed the statistical analysis. HL and HF designed and helped implement the PCASL protocol. HF and AA performed the PCASL analysis. KT supervised the study. All authors contributed to drafting and revising the manuscript.

## Funding

This study was supported by grants from the Science of Learning Institute at Johns Hopkins University and by the NIH/NIDCD through award R01 DC014475 and NIH/NIA through award R01 AG068881 to KT.

## Conflict of Interest

The authors declare that the research was conducted in the absence of any commercial or financial relationships that could be construed as a potential conflict of interest.

## Publisher's Note

All claims expressed in this article are solely those of the authors and do not necessarily represent those of their affiliated organizations, or those of the publisher, the editors and the reviewers. Any product that may be evaluated in this article, or claim that may be made by its manufacturer, is not guaranteed or endorsed by the publisher.
